# Climate, Air Quality, and Emergency Department Use in Northern Germany: Protocol for a Multicenter Register Study

**DOI:** 10.2196/92900

**Published:** 2026-11-10

**Authors:** Tobias Jagomast, Marie Raddatz, Loana Penner, Mareike Lüthgen, Patrick Ristau, Wolfgang Dersch, Sönke Wallis, Friedhelm Sayk, Jörg Metzner, Sebastian Wolfrum, André Calero Valdez, Andrea Baron, Thoralf Kerner, Marcin Grzegorzek, Claudia Schmidtke, Daniel Drömann, Henrik Watz, Maureen Weckmann, Markus Weckmann, Folke Brinkmann, Klaas F Franzen

**Affiliations:** 1 Medical Clinic III, University Hospital Schleswig-Holstein, Ratzeburger Allee 160, Lübeck, 23562, Germany, +4945150075562; 2 Department of Pediatrics, University Hospital Schleswig-Holstein, Lübeck, Germany; 3 Faculty of Applied Health Sciences, Deggendorf Institute of Technology, Deggendorf, Germany; 4 Schön Klinik, Neustadt (Holst.), Germany; 5 Internal Medicine, DRK Hospital Ratzeburg, Ratzeburg, Germany; 6 Sana Kliniken Lübeck, Lübeck, Germany; 7 Emergency Room, University Hospital Schleswig-Holstein, Lübeck, Germany; 8 Institute of Multimedia and Interactive Systems, University of Lübeck, Lübeck, Germany; 9 Department of Anesthesiology, Intensive Care Medicine, Emergency Medicine, Pain and Palliative Therapy, Asklepios Klinikum Harburg, Hamburg, Germany; 10 Asklepios Campus Hamburg, Asklepios Medical School GmbH, Hamburg, Germany; 11 Institute of Medical Informatics, University of Lübeck, Lübeck, Germany; 12 German Research Center for Artificial Intelligence (DFKI), Lübeck, Germany; 13 Airway Research Center North, German Center for Lung Research (DZL), Großhansdorf, Germany

**Keywords:** climate change, emergency department, temperature extremes, air pollution, distributed lag nonlinear models, vulnerable populations, multicenter study, maritime climate, health system preparedness, environmental epidemiology

## Abstract

**Background:**

Climate change threatens population health through temperature extremes, air pollution, and altered infectious disease patterns. Emergency departments (EDs) are sensitive indicators of climate-related health impacts; however, comprehensive multicenter investigations integrating meteorological factors, air quality, and infectious disease surveillance across different health care provision levels remain scarce in Germany, particularly in maritime climate regions.

**Objective:**

This study aims to characterize the impact of meteorological factors, air quality, and infectious disease dynamics on ED presentations across multiple health care facilities and care provision levels in northern Germany over an 11-year period.

**Methods:**

This retrospective, multicenter, observational study will examine ED presentations across 4 facilities representing different care provision levels in the greater Lübeck area and southeastern Schleswig-Holstein, Germany, from January 2014 to December 2024. Clinical data, including ICD (*International Classification of Diseases*)–coded diagnoses, patient demographics, and temporal information, will be linked with meteorological data from the German Weather Service, air quality measurements from state environmental monitoring, and infectious disease surveillance from the Robert Koch Institute. The primary outcomes will be daily ED visits overall and stratified by disease category (cardiovascular, respiratory, psychiatric, infectious, renal, neurological, and surgical/traumatic). Distributed lag nonlinear models within a generalized additive modeling framework will be used to characterize temperature-health relationships, accounting for delayed and nonlinear effects, and adjusted for temporal trends, seasonality, and infectious disease incidence. Subgroup analyses will examine vulnerable populations by age, sex, and socioeconomic status.

**Results:**

Project planning and conceptualization began in June 2024, and ethical approval was granted in July 2025. Following approval, clinical data were transferred sequentially across participating sites in July 2025, August 2025, and October 2025. Data from 3 of the 4 participating hospitals have been successfully received, while the final outstanding dataset is expected in the second half of 2026. Meteorological data collection was completed in October 2025 and air quality data in January 2026. While preliminary descriptive statistical analyses have already commenced, inferential statistics will be performed once data collection is fully completed.

**Conclusions:**

This multicenter study will provide systematic evidence on climate-sensitive ED utilization in a maritime climate region of northern Germany. By integrating clinical, meteorological, air quality, and infectious disease data across 4 hospitals of varying care complexity, the study is well positioned to characterize nonlinear and delayed health effects of temperature extremes across a broad spectrum of acute conditions. Results are expected to inform regional health care capacity planning, early warning systems, and public health preparedness, while contributing to the broader evidence base on climate and health.

## Introduction

Climate change has become increasingly evident in recent decades, with Germany experiencing unprecedented warming. The year 2023 marked the warmest year since meteorological records began, primarily attributed to an exceptionally mild winter, while 2020, the second-warmest year, concluded the warmest decade globally [1,2]. Since 2015, the six warmest years on record have been characterized by repeated droughts in the spring. However, 2023 also recorded one of the highest precipitation amounts, resulting in extensive flooding events, particularly in northern Germany [1,2]. Global warming is projected to exceed both the 1.5 °C and 2 °C thresholds during the 21st century, with profound implications for health care systems [3]. High ambient temperatures impose substantial physiological stress through peripheral vasodilation and accelerated respiration, potentially increasing the uptake of circulating toxic particulate matter [4].

Mounting epidemiological evidence demonstrates a robust association between ambient temperature and emergency department (ED) utilization. Numerous studies have documented marked increases in ED presentations during elevated temperatures, encompassing overall visit volumes and specific increases in infectious, cardiovascular, and psychiatric conditions [5-9]. Multiple studies consistently report that extreme heat elevates ED visits across diverse health conditions, including mental health disorders, respiratory, cardiovascular, and injury-related presentations, with effects typically manifesting immediately or within days [10-20]. Population-based studies from northern Germany using prehospital emergency care data have demonstrated significant associations between ambient temperature and cardiovascular emergencies, with both cold and warm temperatures increasing the frequency and severity of cardiovascular events [21,22]. Many studies have described U-shaped- or V-shaped exposure-response relationships, wherein both high and low temperatures increase the number of ED visits relative to moderate temperatures [13,18,23]. While elevated temperatures produce nearly immediate increases in emergency treatments, cold periods generate delayed rises in patient volumes, extending from one week to several weeks, predominantly affecting respiratory presentations [6,14,17,18,23,24]. Beyond cardiovascular conditions, temperature-dependent patterns have also been observed for metabolic emergencies, with reduced frequency of severe hypoglycemic episodes at mild ambient temperatures between 10°C and 20 °C [21], as well as for toxicological emergencies, with significantly higher rates of severe alcohol and drug poisonings during warmer temperatures [25].

These meteorological impacts are not uniformly distributed across the population. Vulnerable subgroups experience disproportionate weather-related health burdens. Children are particularly susceptible to heat due to their elevated risk of dehydration, immature immune systems, and dependence on adults to regulate heat exposure [5,13,14,18]. Similarly, older adults and individuals with preexisting conditions face heightened vulnerabilities [14,18]. Socioeconomic disadvantage has emerged as a significant risk modifier, with disadvantaged and outdoor-working populations experiencing higher burdens following heat periods [7,13,16,26]. The magnitude of temperature-related risk demonstrates substantial regional and seasonal variation, with maritime climates potentially exhibiting different heat-health relationships compared to continental climates [7,13,18].

Beyond temperature extremes, extreme weather events influence health care systems directly through injuries and indirectly through infection transmission and chronic disease exacerbations. Storms, heavy precipitation, and thunderstorms alter pollen concentrations and particulate matter distribution, potentially triggering asthma exacerbations [3,27]. Air pollution caused by greenhouse gases and particulate matter is a major anthropogenic contributor to climate change [3]. Elevated particulate matter concentrations are associated with increased mortality risk and acute ED presentations, with seasonal differences in composition modified by weather phenomena [4,12,28]. In particular, substantial relationships exist between air pollution and symptom onset or disease exacerbation, especially in asthma, chronic obstructive pulmonary disease, and acute respiratory infections [28]. Both particulate matter and air pollution increase cardiovascular and respiratory ED admissions, with documented impacts on pneumonia with septicemia, asthma exacerbations, and cardiovascular events [4,12,28].

While substantial evidence exists regarding temperature-health relationships globally, comprehensive multicenter investigations integrating meteorological factors, air quality measures, infectious disease surveillance, and socioeconomic determinants across different health care provision levels remain scarce, particularly in German health care settings. Regional differences in climate-related ED visits across Europe reflect local climate extremes and population vulnerabilities, with central and eastern European regions experiencing greater cold-related burdens, whereas southern European regions face intensified heat impacts [29-32]. However, northern Germany, with its distinctive maritime climate and specific socioeconomic structure, represents an understudied region in which climate-health relationships may differ from established patterns. Notable exceptions include a series of population-based studies from Hamburg examining temperature-related emergency service utilization under marine west coast climate conditions, which demonstrated distinct seasonal and temperature-dependent patterns for cardiovascular emergencies, cardiac arrests, metabolic emergencies, and acute poisonings [21,22,25,33]. Methodologically, distributed lag nonlinear models (DLNMs) have emerged as the preferred analytical framework for environmental health research, enabling the characterization of delayed and nonlinear exposure-response relationships [14,34]. More recently, Bayesian spatiotemporal approaches such as integrated nested Laplace approximation (INLA) have been proposed as complementary methods, offering flexible modeling of both spatial and temporal dependencies and providing richer inference on the contributions of individual explanatory variables [35-37]. Given the multicenter design and extended temporal scope of the present study, DLNM was selected as the primary analytical framework due to its established application in environmental epidemiology, while the potential of a spatiotemporal extension will be considered in future analyses [34,35,38]. Multicenter studies incorporating facilities across different care provision levels offer enhanced generalizability and enable the assessment of health care system responses to climate-related challenges.

Against this background, the present study aimed to comprehensively investigate the associations between meteorological factors, climatic developments, infectious disease incidence, and ED presentations across multiple facilities representing different care provision levels in the greater Lübeck area and southeastern Schleswig-Holstein, Germany, over an 11-year period spanning pre–COVID-19, peri–COVID-19, and post–COVID-19 pandemic phases.

The study pursues three specific objectives: first, to characterize the associations between meteorological variables, including temperature extremes, heatwaves, cold spells, and precipitation, and daily ED utilization; second, to examine the independent and combined effects of air quality parameters on ED presentations with particular focus on respiratory and cardiovascular disease categories; and third, to assess effect modification by age group, sex, socioeconomic status, and residential population density. Based on existing evidence, we hypothesize that both heat and cold extremes will be associated with increased ED utilization, with heat effects manifesting within days and cold effects exhibiting delayed associations of up to several weeks, and that vulnerable subgroups, including older adults, children, and socioeconomically disadvantaged populations, will demonstrate elevated risks compared to the general patient population.

## Methods

### Study Design

This investigation uses a retrospective, multicenter observational design to examine ED presentations across multiple health care facilities in the greater Lübeck area and southeastern Schleswig-Holstein, Germany. The study period spans January 1, 2014, to December 31, 2024, encompassing an 11-year observation window. This timeframe was deliberately selected to capture temporal dynamics before, during, and after the COVID-19 pandemic, thereby enabling the assessment of both long-term climatic trends and the impact of infection control measures that may have altered health care–seeking behavior and disease transmission patterns. The extended observation period provides sufficient statistical power to detect seasonal variations, lag effects, and nonlinear exposure-response relationships while accounting for temporal confounding. An overview of the overall study workflow is provided in Figure 1.

**Figure 1.**
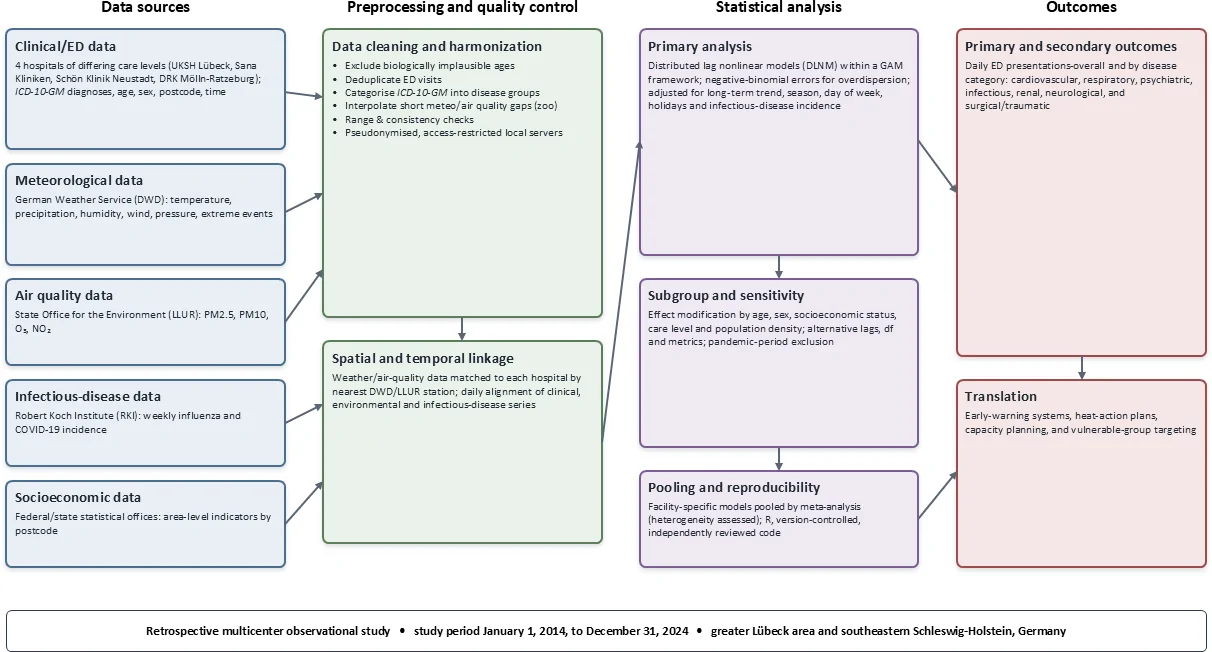
Overview of the study workflow, showing the 5 data sources (clinical/emergency-department, meteorological, air-quality, infectious-disease, and socioeconomic data), the preprocessing and quality-control steps, the spatial and temporal linkage, the statistical-analysis strategy (DLNM within a GAM framework with negative-binomial errors, subgroup and sensitivity analyses, and meta-analytic pooling), and the study outcomes. DRK: Deutsches Rotes Kreuz (German Red Cross); DWD: Deutscher Wetterdienst (German Weather Service); ED: emergency department; GAM: generalized additive model; *ICD-10-GM*: *International Classification of Diseases*, 10th Revision, German Modification; LLUR: Landesamt für Landwirtschaft, Umwelt und ländliche Räume (State Office for Agriculture, Environment and Rural Areas); NO₂: nitrogen dioxide; O₃: ozone; PM2.5: particulate matter ≤2.5 µm in diameter; PM10: particulate matter ≤10 µm in diameter; UKSH: Universitätsklinikum Schleswig-Holstein (University Hospital Schleswig-Holstein).

This protocol is reported in accordance with the RECORD (Reporting of Studies Conducted Using Observational Routinely-Collected Health Data) statement, an extension of the STROBE (Strengthening the Reporting of Observational Studies in Epidemiology) guideline developed specifically for studies using routinely collected health data [39,40]. A completed RECORD checklist is provided as Checklist 1.

### Study Setting and Participating Centers

This study incorporates 4 health care facilities representing different levels of care across the region. The University Hospital Schleswig-Holstein (UKSH) Lübeck serves as a regional maximum care provider, contributing data from both the interdisciplinary and pediatric EDs. Sana Kliniken Lübeck is a large urban hospital serving the metropolitan area. Schön Klinik Neustadt provides specialized cardiovascular and orthopedic care in a midsized coastal town. The DRK Hospital Mölln-Ratzeburg serves a rural catchment area in southeastern Schleswig-Holstein. This diversity in care provision levels, geographic distribution, and catchment area characteristics enhances the generalizability of the findings while enabling the examination of potential differences in climate-health relationships across health care delivery contexts and socioeconomic populations.

### Data Sources

#### Clinical Data

ED data will be extracted from the electronic health record systems of the participating institutions. The inclusion criteria encompass all patients presenting to the EDs during the study period. The data elements include patient age, sex, and residential postal code for demographic and geographic characterization. Clinical information comprises admission and discharge diagnoses coded according to the *ICD-10-GM* (*International Classification of Diseases*, 10th Revision, German Modification). Temporal variables include the admission date and time, discharge date and time, as well as information on whether treatment is outpatient or inpatient. Diagnoses will be categorized into major disease groups, including cardiovascular conditions, respiratory diseases, psychiatric and behavioral disorders, infectious diseases, renal disorders, neurological conditions, and surgical or traumatic presentations, based on *ICD* code chapters and specific diagnostic groupings relevant to climate-health research. As Germany’s hospital reimbursement system continues to mandate the use of *ICD-10-GM* for inpatient billing, all diagnoses across participating institutions will be consistently coded according to *ICD-10-GM*. Consequently, no cross-version harmonization will be required, ensuring coding comparability across the full study period.

Records with biologically implausible age values (<0 or>110 y) will be excluded as probable data-entry errors. Presentations with a missing residential postal code will be retained for overall and diagnosis-specific counts but excluded from spatially stratified analyses (socioeconomic and population-density subgroups). Interfacility transfers identifiable from the admission source will be flagged and, where the same presentation is documented at more than one participating site, counted once at the first presenting facility to avoid double counting. For analyses of overall case volumes, all remaining records will be retained regardless of missing values in individual variables. For analyses requiring specific variables, such as regional stratification by postal code, only cases with complete data for the variable of interest will be included, with analytic sample sizes reported for each analysis separately.

To ensure accurate case counts, duplicate records arising from multiple diagnostic entries per patient visit were identified and consolidated. Each visit was assigned a unique identifier based on a combination of consistent patient-level and visit-level variables, and duplicate entries sharing the same identifier were merged into a single record prior to analysis.

#### Meteorological Data

Meteorological parameters will be obtained from the German Weather Service (Deutscher Wetterdienst, DWD), which maintains high-quality weather-monitoring networks across Germany. The daily meteorological variables will include minimum, maximum, and mean ambient temperatures, precipitation amounts, relative humidity, wind speed, and atmospheric pressure. Additionally, extreme weather events, such as heatwaves, cold spells, heavy precipitation events, and severe storms, will be identified based on established meteorological criteria. Weather station data will be spatially matched to participating hospitals, with appropriate interpolation methods applied, where necessary, to ensure a representative exposure assessment for each facility’s catchment area.

To analyze daily emergency visits, meteorological and air quality data will be linked to each treating hospital based on its geographic proximity to the nearest DWD monitoring station.

Missing values in meteorological variables will be addressed through linear interpolation for gaps of up to 2 consecutive days, using the *na.approx* function from the *zoo* package in R (R Foundation for Statistical Computing). Larger gaps will be retained as missing and excluded from the respective analyses.

#### Air Quality Data

Air quality measurements will be acquired from monitoring stations operated by the State Office for the Environment of Schleswig-Holstein (Landesamt für Umwelt Schleswig-Holstein). The parameters will include fine particulate matter with an aerodynamic diameter ≤2.5 µm (PM2.5) or ≤10 µm (PM10), ozone (O_₃_), and nitrogen dioxide (NO_₂_). These pollutants will be selected based on their documented health impacts and the availability of continuous monitoring data throughout the study region. Pollutant concentrations will be assigned to ED visits based on the geographic proximity of monitoring stations to each participating hospital. Missing values will be handled through linear interpolation as described in the *Meteorological Data* section.

#### Infectious Disease Surveillance Data

Weekly incidence data for notifiable infectious diseases will be obtained from the Robert Koch Institute, Germany’s national public health institute. The primary focus will be on influenza and COVID-19, given their substantial impact on ED utilization and potential interactions with weather-related presentations. Incidence rates will be expressed per 100,000 population according to the reporting requirements of the German Infection Protection Act (Infektionsschutzgesetz). These data enable control for temporal confounding by infectious disease epidemics and the assessment of the potential synergistic effects between the infectious disease burden and meteorological factors.

#### Socioeconomic Data

Socioeconomic characteristics will be assessed at the postal code level using patients’ residential information. Regional socioeconomic indicators will be derived from official statistics provided by federal and state statistical offices, enabling the classification of catchment areas by socioeconomic status. This geographic linkage approach, while ecological in nature, permits the examination of socioeconomic disparities in weather-related ED utilization without requiring individual-level socioeconomic data that may be incomplete in ED records.

### Outcomes

The primary outcome will be the daily count of ED presentations, stratified by disease category. The secondary outcomes include category-specific presentations of cardiovascular, respiratory, psychiatric, infectious, renal, neurological, and surgical/traumatic conditions. Additional analyses will examine presentations for specific conditions that are particularly relevant to climate and air quality, including asthma and chronic obstructive pulmonary disease exacerbations, acute respiratory infections, acute coronary syndromes, heart failure, heat-related illnesses, and cold-related conditions. Age-stratified analyses will focus on toddlers (<5 y), children/adolescents (5‐18 y), working-age adults (18‐65 y), and older adults (≥65 y) to identify age-specific vulnerabilities.

### Statistical Analysis

#### Descriptive Analysis

Initial descriptive analyses will characterize the study population, temporal trends in ED utilization, and the distribution of meteorological and air quality variables. Time series plots will visualize temporal patterns, seasonal variations, and potential structural breaks corresponding to the COVID-19 pandemic. Descriptive statistics will be used to summarize patient demographics, disease categories, and exposure distributions across the study period and participating facilities.

#### Primary Analysis

The primary analytical approach will combine DLNM within a generalized additive model (GAM) framework, which has become the reference standard for environmental epidemiology, given its capacity to simultaneously model nonlinear exposure-response relationships and delayed effects [34,41,42]. While Bayesian spatiotemporal approaches such as INLA offer complementary advantages, DLNM was selected as the primary framework given its established application in environmental epidemiology and its capacity to simultaneously capture nonlinear exposure-response and lag-response dimensions within a unified framework. This approach addresses the reality that the health impacts of temperature and air pollution may not follow linear dose-response relationships and may manifest over multiple days or weeks following exposure [34,37,43]. Cross-basis functions will be constructed to represent both the exposure-response and lag-response dimensions. For temperature, natural cubic splines will model nonlinear relationships, with degrees of freedom selected based on the Akaike information criterion (AIC) model fit criteria and subject-matter considerations [34]. Lag structures will extend up to 21 days for temperature effects, with longer lags examined for cold exposure, given the documented delayed impacts on respiratory presentations [6,13,24]. For air pollutants, shorter lag structures of up to five days will be applied consistent with the more immediate onset of pollutant-related health effects [4,44,45]. Separate models will be fitted for heat and cold effects relative to the minimum morbidity temperature (MMT) identified from the data.

Negative binomial regression models will be employed to account for overdispersion in daily ED count data, providing a fully specified likelihood framework and enabling AIC-based model comparison for the selection of optimal spline degrees of freedom. Models will be adjusted for long-term trends using natural splines of time, day of week effects, public holidays, and seasonal patterns [34]. Infectious disease incidence will be included as a time-varying covariate to control for confounding factors during epidemic periods. Interaction terms will be examined to assess the effect of age group, sex, and socioeconomic status. Facility-specific models will be fitted initially, with meta-analytical approaches used to pool estimates across centers while assessing heterogeneity.

For air pollution analyses, similar DLNM frameworks will be applied to model the relationships between particulate matter and gaseous pollutants and respiratory and cardiovascular presentations [28,46]. Multipollutant models will be examined where data permit to assess the independent effects of different pollutants. Potential synergistic effects between temperature and air pollution will be explored using interaction terms and stratified analyses [4].

#### Reproducibility

All analyses will be conducted in R, using the *dlnm* package for the construction and fitting of DLNMs with negative binomial errors, alongside the *mgcv* package for GAM fitting, and the *meta* package for pooling of facility-specific estimates. Analysis scripts will be available from the corresponding author upon reasonable request.

#### Subgroup and Sensitivity Analyses

Subgroup analyses will systematically examine effect modification by age group (children, working-age adults, and older adults 65≥ y), sex, socioeconomic status, and care provision level. Sensitivity analyses will assess the robustness of the findings to alternative model specifications, including varying degrees of freedom for spline functions modeling long-term trends, alternative lag structures for temperature (14 vs 21 d) and air pollutants (3 vs 5 d), exclusion of pandemic periods, and alternative temperature metrics (minimum, maximum, or mean daily temperature).

Facility-specific estimates will be examined individually to assess heterogeneity in exposure-response relationships across participating centers. To assess the potential influence of missing meteorological values, sensitivity analyses will compare results obtained using linearly interpolated weather data against those based on complete observed measurements only.

Additional subgroup analyses will stratify patients by residential population density, classified into low, medium, and high density, based on residential postal code. Separate models will be fitted for each density category to examine whether associations between meteorological exposures and ED utilization differ across population density groups.

To evaluate the influence of the spatial assignment of environmental exposures, sensitivity analyses will compare the primary nearest-station approach against alternative assignment strategies, including inverse-distance-weighted interpolation across the 2 or 3 closest monitoring stations and, where station density permits, area-level averaging within the hospital catchment. Effect estimates obtained under these alternative matching schemes will be compared with the primary specification to assess the robustness of exposure-response relationships to exposure misclassification arising from station choice.

### Sample Size and Statistical Power

Based on previous similar investigations [4,6] and preliminary data from the participating centers, we anticipate approximately 800,000 to 1,000,000 ED presentations across the 4 facilities over the 11-year study period. This substantial sample size provides excellent statistical power to detect modest effect sizes, even in subgroup analyses. For example, with daily counts averaging 200 to 250 presentations across facilities, we have >90% power to detect relative risk increases of 5%‐10% per 10 °C temperature change at an *α* level of .05, consistent with effect sizes reported in comparable studies [5,7,8]. The multicenter design enhances the power to examine heterogeneity, while the extended time series enables robust control for seasonal and long-term trends [34].

### Data Management and Quality Control

Data extraction and harmonization protocols will standardize the information across participating centers, addressing potential differences in coding practices, data completeness, and documentation standards. A centralized database will be established with quality control procedures, including range checks, logical consistency checks, and verification of temporal sequences. Missing data patterns will be systematically characterized, and regular data quality reports will be generated to identify anomalies requiring investigation. All data linkage procedures, including the temporal and spatial matching of environmental exposures to health outcomes, will be documented and validated.

All data will be stored on password-protected local and institutional servers of the University Hospital Lübeck (UKSH Lübeck), with access restricted to authenticated members of the study group. To minimize the risk of reidentification of pseudonymized patient data through cross-referencing covariables such as postal code, age, and sex, individual-level data will not be shared beyond the authorized study team. Results will be released exclusively in aggregated form as daily case counts.

Analytical code will be developed in R and stored locally, with a transfer to a version-controlled repository planned to ensure transparency and reproducibility. Code will be reviewed by an independent member of the study team to minimize the risk of analytical errors. Analysis scripts will be made available upon reasonable request.

Analytical code will be maintained under Git-based version control in a private institutional repository, with access secured via multifactor authentication and regular encrypted backups. Analytical steps will be documented in reproducible notebooks integrating code, results, and narrative, so that tables and figures are generated directly from the analysis without manual transfer. Code will be independently re-executed in a clean computing environment by a study-team member not involved in the original implementation, and identified issues will be tracked and resolved through the repository’s issue-tracking system.

### Ethical Considerations

This retrospective study will use pseudonymized routine clinical data without individual patient contact. The legal basis for data processing is established under Section 38 of the State Hospital Act (Landeskrankenhausgesetz, LKHG) of Schleswig-Holstein [47] (effective since July 5, 2024), which permits further processing of patient data for research purposes, provided that the use of these data does not compromise the treatment of patients and that the fundamental principles of the General Data Protection Regulation and the State Data Protection Act are adhered to. Ethical approval was granted by the responsible ethics committee in July 2025.

In accordance with Section 6 of the Health Data Usage Act (Gesundheitsdatennutzungsgesetz; [48]), lawfully stored data may be further processed for medical research purposes, with pseudonymization applied at the earliest possible stage of data processing. Prior to data transfer, all direct identifiers, including patient identification numbers, were removed by the providing institutions.

Ethical approval was obtained from the ethics committee of the University of Lübeck (AZ 2025‐269), including review of the inclusion of vulnerable population groups. The study protocol was reviewed by the institutional data protection officers at all participating facilities. Data-sharing agreements between institutions specify the permitted uses, security requirements, and duration of data retention. Results will be reported in aggregate form only, with measures to prevent the potential identification of individuals in small cells (ie, groups with fewer than 5 cases). The inclusion of vulnerable populations, including children, individuals with chronic conditions, and socioeconomically disadvantaged groups, is scientifically necessary, given their documented elevated risk of weather-related health impacts. Exclusion of these groups would bias the results and potentially perpetuate health inequities by limiting the evidence for targeted interventions.

This study will be conducted in accordance with Article 35 of the Declaration of Helsinki (DRKS-ID: DRKS00037454). The ethics committee waived the need for ethical approval and patient consent for the collection, analysis, and publication of retrospectively obtained anonymized data for this noninterventional study.

## Results

### Study Status

The study schedule and the current status of data acquisition and analysis are summarized in Table 1.

**Table 1.** Study schedule and current status of data acquisition and analysis.

Milestone	Date	Status
Project planning and conceptualization	June 2024	Completed
Ethics approval (University of Lübeck, AZ 2025‐269)	July 2025	Granted
Infectious-disease data acquisition (Robert Koch Institute)	September 2025	Completed
Clinical data acquisition, sites 1‐3	July-October 2025	Received
Meteorological data acquisition (German Weather Service)	October 2025	Completed
Air-quality data acquisition (State Office for the Environment)	January 2026	Completed
Clinical data acquisition, site 4	Second half of 2026	Anticipated
Descriptive analyses	2026	Commenced
Data integration, inferential analyses, and reporting	2026‐2027	Planned

Project planning and conceptualization commenced in June 2024.

Clinical data collection was conducted sequentially across the 4 participating hospitals. Data from 3 of the 4 sites were successfully received in July 2025, August 2025, and October 2025, respectively. The fourth and final dataset is anticipated in the second half of 2026. No deviations from the originally planned study protocol have occurred.

Meteorological data from the German Weather Service were obtained in October 2025, covering the full study period from January 2014 to December 2024. Complete air quality measurements from state environmental monitoring were received in January 2026. Infectious disease surveillance data from the Robert Koch Institute were obtained in September 2025.

Across the three datasets received, a preliminary case count indicates that 601,734 ED presentations have been recorded. Baseline characteristics of included patients, including age distribution, sex, and *ICD*-coded primary diagnoses, will be described in full upon completion of data collection. No major deviations from the planned inclusion criteria have been identified in the datasets received.

Descriptive statistical analyses of the available data have been initiated. Inferential statistical modeling will be performed upon receipt and integration of all data sources.

### Dissemination

The study findings will be disseminated through peer-reviewed publications in international journals focusing on environmental health, public health, and emergency medicine. The results will be presented at relevant scientific conferences. A lay summary will be prepared for communication with the participating institutions, public health authorities, and the general public. Individual participating facilities will receive site-specific reports to inform local adaptation plans. Data will be made available through recognized research data repositories following appropriate embargo periods and in compliance with data protection regulations, with individual-level data being pseudonymized and aggregated as necessary.

## Discussion

### Strengths

This investigation has several methodological strengths that enhance the validity and generalizability of the anticipated findings. The multicenter design incorporating four health care facilities across different care provision levels and geographic locations provides diverse patient populations and health care delivery contexts, enhancing external validity beyond single-center studies. The 11-year observation period offers substantial statistical power while enabling robust control of temporal trends, seasonal patterns, and long-term climatic shifts [4,6]. Critically, the study period encompassed the prepandemic, pandemic, and postpandemic phases, permitting the assessment of how infection control measures and altered health care–seeking behaviors may have modified climate-health relationships.

Comprehensive exposure assessment integrates multiple meteorological parameters, air quality measures, and infectious disease surveillance data, enabling the examination of independent, interactive, and synergistic effects [3,4]. The use of DLNM represents a widely adopted analytical framework in environmental epidemiology [14,34], enabling the characterization of both immediate and delayed health impacts while accounting for nonlinear exposure-response relationships [14,18]. The inclusion of facilities serving diverse socioeconomic populations combined with postal code–level geographic information enables the investigation of health inequities in climate-related ED utilization [7,16,26,49], addressing an important gap in the German context. Finally, the focus on northern Germany, with its distinctive maritime climate, contributes evidence from an understudied region where heat-health and cold-health relationships may differ from continental climate zones that have dominated European climate-health research [30-32]. This investigation builds upon prior population-based studies from the Hamburg metropolitan area that have established temperature-dependent patterns for various emergency conditions under marine west coast climate conditions [21,22,25,33], extending this evidence base through multicenter, multifacility analysis of ED presentations.

### Limitations

Several limitations warrant consideration when interpreting the study findings. The retrospective observational design precludes causal inference in the strict experimental sense, although the temporal nature of exposure-outcome relationships and the use of time-series methods with appropriate confounder adjustment provide evidence for causal associations following the Bradford Hill criteria [34]. The ecological approach to socioeconomic characterization using postal code-level data introduces potential ecological fallacy, as within-area heterogeneity cannot be captured, and individual-level socioeconomic circumstances may differ substantially from area-level averages [26].

Weather and air quality exposure assignment based on fixed monitoring stations may introduce measurement errors, particularly for individuals residing at greater distances from monitoring locations or spending substantial time in locations different from their residence [8]. This classical exposure measurement error would typically bias effect estimates toward the null hypothesis, suggesting that true associations may be stronger than those observed. The lack of individual-level data on factors such as housing characteristics, air conditioning availability, occupational exposures, and behavioral adaptations limits the ability to identify specific mechanisms underlying observed associations or to characterize highly vulnerable individuals within broader demographic groups [5,7].

The generalizability of the findings to other German regions or international contexts requires careful consideration, given the potential differences in population characteristics, health care system organization, climate adaptation measures, and baseline climate conditions [31,32]. The focus on ED presentations rather than community-level disease occurrence means that the findings reflect health care utilization conditional on seeking emergency care, potentially missing milder cases managed in primary care or not seeking medical attention. Changes in health care access, insurance coverage, or facility capacity during the study period may have confounded the temporal trends in presentation rates. Finally, while the study examined multiple disease categories, residual confounding by unmeasured factors, including co-occurring environmental exposures, population mobility patterns, or temporal changes in diagnostic coding practices, cannot be entirely excluded despite careful analytical approaches.

### Expected Impact and Implications

This investigation is expected to generate important evidence with implications for public health preparedness, health care system planning, and climate adaptation strategies in northern Germany and comparable maritime climate regions [3]. By quantifying the associations between meteorological factors and ED utilization across multiple disease categories and patient subgroups, the findings will inform the development of targeted early warning systems and preventive interventions [16,50,51]. The identification of vulnerable populations and high-risk periods will enable more efficient allocation of public health resources and clinical capacity during extreme weather events [52,53].

A comprehensive assessment of the impact of air quality alongside meteorological factors will contribute to integrated environmental health strategies addressing both climate change mitigation and adaptation [3,28]. Evidence of synergistic effects between temperature, air pollution, and infectious disease incidence would support the need for coordinated surveillance and intervention programs [4]. The multicenter design encompassing different care provision levels will inform health care system resilience planning, highlighting which facility types and patient populations experience the greatest climate-related pressures.

From a scientific perspective, this study advances methodological approaches to multicenter climate-health research in Germany while contributing to the limited evidence base regarding maritime climate regions in Europe [29,31]. The inclusion of the COVID-19 pandemic period provides unique opportunities to examine how infectious disease epidemics and associated control measures modify climate-health relationships, with potential relevance for future pandemic preparedness. The findings may inform the revision of clinical guidelines for managing heat- and cold-related illnesses [14], the development of facility-level heat action plans [54], and the integration of climate considerations into ED operations and staffing models.

More broadly, this study will support evidence-based climate change adaptation in the health sector, contributing to national and international climate-health assessments [3,32]. By demonstrating the feasibility and value of linking routine health data with environmental monitoring for climate-health surveillance, this study may catalyze similar investigations in other regions and support the establishment of ongoing surveillance systems [16]. The open data sharing approach will facilitate secondary analyses, meta-analyses, and methodological comparisons, advancing the field of climate-health research.

### Conclusions

Climate change poses substantial and growing challenges to emergency health care delivery through multiple pathways, including temperature extremes, air pollution, and altered infectious disease patterns [3]. This comprehensive multicenter investigation will characterize these relationships in the context of northern Germany’s distinctive maritime climate, identify vulnerable populations, and quantify health system impacts over an 11-year period spanning the COVID-19 pandemic. By integrating rigorous environmental epidemiology methods [34] with routine health care data across multiple facilities and care provision levels, this study will generate actionable evidence to inform climate adaptation strategies, public health interventions, and health care system preparedness [16,51]. The findings will contribute to protecting population health in the face of accelerating climate change while advancing the scientific understanding of climate-health relationships in understudied European regions [31,32].

## Supplementary material

10.2196/92900 Checklist 1 RECORD checklist.

## Abbreviations

AIC: Akaike information criterion
DLNM: distributed lag nonlinear model
DRK: Deutsches Rotes Kreuz (German Red Cross)
DWD: Deutscher Wetterdienst (German Weather Service)
ED: emergency department
GAM: generalized additive model
*ICD*: *International Classification of Diseases*
*ICD-10-GM*: *International Classification of Diseases*, 10th Revision, German Modification
INLA: integrated nested Laplace approximation
MMT: minimum morbidity temperature
PM10: particulate matter ≤10 µm in diameter
PM2.5: particulate matter ≤2.5 µm in diameter
RECORD: Reporting of Studies Conducted Using Observational Routinely-Collected Health Data
STROBE: Strengthening the Reporting of Observational Studies in Epidemiology
UKSH: Universitätsklinikum Schleswig-Holstein (University Hospital Schleswig-Holstein)
