# Supplementary material for: Climate, Air Quality, and Emergency Department Use in Northern Germany: Protocol for a Multicenter Register Study
Source: JMIR Res Protoc. 2026 Nov 10;15:e92900. doi: 10.2196/92900 (PMC13750629; doi:10.2196/92900)
Supplement: Supplementary file 1 [file resprot-v15-e92900-s001.docx]

**Multimedia Appendix 1. Completed RECORD checklist**

Climate, Air Quality, and Emergency Department Utilization in Northern Germany: Protocol for a Multi-Center Study (2014–2024)

| **Item No.** | **STROBE items** | **Location in manuscript** | **RECORD items** | **Location in manuscript** |
| --- | --- | --- | --- | --- |
| **Title and Abstract** | | | | |
| **1** | (a) Indicate the study’s design with a commonly used term in the title or the abstract.  (b) Provide in the abstract an informative and balanced summary of what was done and what was found. | Title (“Protocol for a Multi-Center Study”); Abstract (Methods: “retrospective, multicentre, observational study”) | RECORD 1.1: The type of data used should be specified in the title or abstract. When possible, the name of the databases used should be included.  RECORD 1.2: If applicable, the geographic region and timeframe within which the study took place should be reported in the title or abstract.  RECORD 1.3: If linkage between databases was conducted for the study, this should be clearly stated in the title or abstract. | 1.1 Abstract (Methods): clinical/ED, meteorological (DWD), air-quality and infectious-disease (RKI) data named. 1.2 Title (“Northern Germany … 2014–2024”); Abstract (Methods): Lübeck area & SE Schleswig-Holstein, Jan 2014–Dec 2024. 1.3 Abstract (Methods): clinical data “will be linked with” meteorological, air-quality and surveillance data. |
| **Introduction** | | | | |
| **2** | Explain the scientific background and rationale for the investigation being reported. | Introduction (paragraphs 1–5) |  |  |
| **3** | State specific objectives, including any prespecified hypotheses. | Introduction (final paragraph: three specific objectives and hypotheses); Abstract (Objectives) |  |  |
| **Methods** | | | | |
| **4** | Present key elements of study design early in the paper. | Methods → Study Design |  |  |
| **5** | Describe the setting, locations, and relevant dates, including periods of recruitment, exposure, follow-up, and data collection. | Methods → Study Design and Study Setting and Participating Centres; Results (data-collection dates) |  |  |
| **6** | (a) Cohort study — Give the eligibility criteria, and the sources and methods of selection of participants; describe methods of follow-up. Case-control / cross-sectional — as applicable.  (b) For matched studies, give matching criteria and numbers of exposed/unexposed (or controls per case). | (a) Methods → Data Sources (Clinical Data): inclusion criteria and case selection. (b) Not applicable (unmatched, time-series design). | RECORD 6.1: The methods of study population selection (such as codes or algorithms used to identify subjects) should be listed in detail. If this is not possible, an explanation should be provided.  RECORD 6.2: Any validation studies of the codes or algorithms used to select the population should be referenced. If validation was conducted for this study and not published elsewhere, detailed methods and results should be provided.  RECORD 6.3: If the study involved linkage of databases, consider use of a flow diagram or other graphical display to demonstrate the data linkage process, including the number of individuals with linked data at each stage. | 6.1 Methods → Clinical Data: ED presentations selected by EHR extraction and ICD-10-GM diagnosis groups; exclusion of implausible ages and duplicate consolidation described. 6.2 Not applicable — nationally standardized ICD-10-GM coding used; no separate code-validation study [authors to confirm]. 6.3 Data-linkage process described in Methods → Meteorological Data (spatial-temporal matching); overall workflow shown in Figure 1. Linked-record counts to be reported upon completion. |
| **7** | Clearly define all outcomes, exposures, predictors, potential confounders, and effect modifiers. Give diagnostic criteria, if applicable. | Methods → Data Sources; Outcomes; Statistical Analysis (Primary Analysis) | RECORD 7.1: A complete list of codes and algorithms used to classify exposures, outcomes, confounders, and effect modifiers should be provided. If these cannot be reported, an explanation should be provided. | Disease groupings defined in Methods → Clinical Data and Outcomes. A complete ICD-10-GM code list will be provided as a supplementary appendix [to be added]. |
| **8** | For each variable of interest, give sources of data and details of methods of assessment (measurement). Describe comparability of assessment methods if there is more than one group. | Methods → Data Sources (Clinical, Meteorological, Air Quality, Infectious Disease Surveillance, Socioeconomic) |  |  |
| **9** | Describe any efforts to address potential sources of bias. | Methods → Statistical Analysis (confounder adjustment; Subgroup and Sensitivity Analyses); Data Management and Quality Control; Discussion → Limitations |  |  |
| **10** | Explain how the study size was arrived at. | Methods → Sample Size and Statistical Power |  |  |
| **11** | Explain how quantitative variables were handled in the analyses. If applicable, describe which groupings were chosen, and why. | Methods → Statistical Analysis (Primary Analysis: cross-basis functions, splines, lag structure); Outcomes (age strata; population-density groups) |  |  |
| **12** | (a) Describe all statistical methods, including those used to control for confounding.  (b) Describe any methods used to examine subgroups and interactions.  (c) Explain how missing data were addressed.  (d) If applicable, explain how loss to follow-up / matching / sampling strategy was addressed.  (e) Describe any sensitivity analyses. | (a) Methods → Primary Analysis (DLNM/GAM, negative-binomial, AIC). (b) Subgroup and Sensitivity Analyses. (c) Data Sources (linear interpolation, zoo); Clinical Data (missing-data handling); Subgroup and Sensitivity Analyses (interpolation sensitivity). (d) Not applicable (no individual follow-up/matching). (e) Subgroup and Sensitivity Analyses. |  |  |
| **—** | **Data access and cleaning methods** |  | RECORD 12.1: Authors should describe the extent to which the investigators had access to the database population used to create the study population.  RECORD 12.2: Authors should provide information on the data cleaning methods used in the study. | 12.1 Methods → Data Management and Quality Control (access restricted to authenticated study-group members); Ethical Considerations. 12.2 Methods → Data Management and Quality Control; Clinical Data; Meteorological Data (range/consistency checks, de-duplication, interpolation). |
| **—** | **Linkage** |  | RECORD 12.3: State whether the study included person-level, institutional-level, or other data linkage across two or more databases. The methods of linkage and methods of linkage quality evaluation should be provided. | Methods → Meteorological Data (facility-level spatial-temporal linkage of clinical, environmental and surveillance data by nearest station); Data Management and Quality Control (linkage documented and validated). |
| **Results (study protocol — analytic results to be reported upon completion)** | | | | |
| **13** | (a) Report the numbers of individuals at each stage of the study (potentially eligible, examined, eligible, included, analysed).  (b) Give reasons for non-participation at each stage.  (c) Consider use of a flow diagram. | (a) Results: preliminary count of 601,734 presentations across three received datasets; full numbers upon completion. (b) To be reported upon completion. (c) Workflow shown in Figure 1; participant flow diagram upon completion. | RECORD 13.1: Describe in detail the selection of the persons included in the study (i.e., study population selection) including filtering based on data quality, data availability and linkage. The selection of included persons can be described in the text and/or by means of the study flow diagram. | Methods → Clinical Data (inclusion/exclusion, plausibility filtering, de-duplication); Results (datasets received). |
| **14** | (a) Give characteristics of study participants and information on exposures and potential confounders.  (b) Indicate the number of participants with missing data for each variable of interest.  (c) Cohort study — summarise follow-up time. | Results: baseline characteristics (age, sex, ICD-coded diagnoses) to be described upon completion of data integration. |  |  |
| **15** | Report numbers of outcome events or summary measures (over time / by exposure category), per design. | Not applicable at protocol stage; outcomes defined in Methods → Outcomes. |  |  |
| **16** | (a) Give unadjusted and, if applicable, confounder-adjusted estimates and their precision; state which confounders were adjusted for and why.  (b) Report category boundaries when continuous variables were categorized.  (c) If relevant, translate relative into absolute risk. | Not applicable at protocol stage (no analytic results reported). |  |  |
| **17** | Report other analyses done — e.g., subgroups, interactions, sensitivity analyses. | Prespecified in Methods → Subgroup and Sensitivity Analyses; results upon completion. |  |  |
| **Discussion** | | | | |
| **18** | Summarise key results with reference to study objectives. | Discussion → Strengths; Expected Impact and Implications |  |  |
| **19** | Discuss limitations of the study, taking into account sources of potential bias or imprecision; discuss both direction and magnitude of any potential bias. | Discussion → Limitations | RECORD 19.1: Discuss the implications of using data that were not created or collected to answer the specific research question(s). Include discussion of misclassification bias, unmeasured confounding, missing data, and changing eligibility over time, as they pertain to the study being reported. | Discussion → Limitations (ecological fallacy, exposure measurement error, lack of individual-level data, residual confounding, changes in diagnostic coding practices over time). |
| **20** | Give a cautious overall interpretation of results considering objectives, limitations, multiplicity of analyses, results from similar studies, and other relevant evidence. | Discussion → Expected Impact and Implications; Conclusion |  |  |
| **21** | Discuss the generalisability (external validity) of the study results. | Discussion → Strengths (multicentre, care-level diversity); Limitations (generalizability) |  |  |
| **Other Information** | | | | |
| **22** | Give the source of funding and the role of the funders for the present study and, if applicable, for the original study on which the present article is based. | Funding (end matter) |  |  |
| **—** | **Accessibility of protocol, raw data, and programming code** |  | RECORD 22.1: Authors should provide information on how to access any supplemental information such as the study protocol, raw data, or programming code. | Data availability statement; Methods → Reproducibility (analysis scripts available on request); Dissemination (DRKS registration: DRKS00037454). |

Reference: Benchimol EI, Smeeth L, Guttmann A, Harron K, Moher D, Petersen I, Sørensen HT, von Elm E, Langan SM; RECORD Working Committee. The REporting of studies Conducted using Observational Routinely-collected health Data (RECORD) Statement. PLoS Med. 2015;12(10):e1001885.

*The RECORD checklist is protected under the Creative Commons Attribution (CC BY) license.*
